# Marine-Derived Steroids for Cancer Treatment: Search for Potential Selective Glucocorticoid Receptor Agonists/Modulators (SEGRAMs)

**DOI:** 10.3390/md23100399

**Published:** 2025-10-14

**Authors:** Ekaterina M. Zhidkova, Ekaterina D. Savina, Ekaterina A. Yurchenko, Ekaterina A. Lesovaya

**Affiliations:** 1 Department of Chemical Carcinogenesis, Institute of Carcinogenesis, N.N. Blokhin National Medical Research Center for Oncology, Kashirskoe Shosse 24-15, Moscow 115478, Russiakaty.dm.savina@gmail.com (E.D.S.); 2 G.B. Elyakov Pacific Institute of Bioorganic Chemistry, Far Eastern Branch of the Russian Academy of Sciences, 159 Prospect 100-Letiya Vladivostoka, Vladivostok 690022, Russia; dminae@mail.ru; 3 Institute of Medicine, Peoples’ Friendship University of Russia, Miklukho-Maklaya St. 6, Moscow 117198, Russia; 4 Faculty of Oncology, I.P. Pavlov Ryazan State Medical University, Vysokovol’tnaya St. 9, Ryazan 390026, Russia

**Keywords:** marine-derived steroids, anti-cancer therapy, anti-inflammatory effects, glucocorticoid, selective glucocorticoid receptor agonists

## Abstract

Steroids, particularly glucocorticoids, are essential components of cancer treatment for both hematological malignancies and solid tumors. The adverse effects of standard steroid-based drugs have forced drug discovery research to develop chemotherapeutics with a more selective mechanism of action and an improved therapeutic index. Steroids of natural origin and their analogs are a significant source of novel molecules with a wide spectrum of biological activities. In this review, we aimed to analyze marine-derived steroids and their anti-cancer activity. Moreover, we specifically discussed molecules with not only anti-cancer but also anti-inflammatory activities that could potentially mimic the effects of glucocorticoids. We hypothesized that several of the reviewed compounds could exhibit affinity to the glucocorticoid receptor, and possess the properties of selective glucocorticoid receptor agonists/modulators with increased therapeutic activity and decreased side effects. The review is based on the literature available in the PubMed, Cochrane, and ClinicalTrials.gov databases and covers the period from 1986 to 2025. The keywords used were “steroids”, “cancer”, and “marine-derived steroids”. The second iteration of the literature search included the keywords “selective glucocorticoid receptor agonists”, “marine-derived”, and “cancer”. In silico calculations of several marine-derived compounds were performed to support the hypothesis based on the literature data.

## 1. Introduction

Cancer pathologies are characterized by the uncontrolled proliferation of transformed cells, replacement of normal tissues, and invasion and metastasis to adjacent and distant organs. The pleiotropic mechanisms of cell transformation, including cell cycle disruption, attenuation of apoptosis, aberrant signaling, neoangiogenesis, invasion, and changes in tumor epigenetics and microenvironment, require a multifactorial treatment strategy [1,2,3]. Targeted cancer therapy is crucially dependent on the identification of specific biomarkers, which are frequently associated with the activation of alternative signaling pathways and, consequently, the development of pharmacological resistance [4,5]. Multitargeted therapies could be a more effective alternative, using either drug combinations or a single drug with multiple targets [6]. There are several options for multitargeted drug exploration: (1) the design of novel molecules with a number of targets (i.e., low-molecular-weight multikinase inhibitors) [6,7,8,9,10,11]; (2) repurposing of registered and marketed drugs after a detailed study of the mechanism of action and revelation of novel targets (i.e., thalidomide, rapamycin) [12,13,14,15,16,17,18,19]; and (3) the use of biologically active compounds of natural origin and their secondary metabolites (i.e., polyphenols, flavonoids) with multiple targets and usually mild and reversible effects [20,21,22,23,24,25,26,27,28].

Steroids can be discussed as multitargeted molecules that can be applied and repurposed in various ways, including in cancer treatment. Structurally, steroids are hydrophobic molecules that are biosynthesized from cholesterol and can also be obtained from numerous terrestrial and marine sources [29,30,31]. Physiologically, steroid hormones are responsible for sex differentiation and reproduction (androgens, estrogens, progestogens), metabolism and immunity (glucocorticoids, GC), homeostasis, blood volume and electrolyte maintenance (mineralocorticoids), and calcium absorption (calciferols) [32,33]. In cancer therapy, steroids are used because of their binding to specific receptors overproduced in particular tissues. Ligand-dependent receptors activate transcription factors, which regulate the transcription of dependent genes, resulting in changes in cancer cell proliferation [34,35,36,37]. Moreover, competitive synthetic ligands for steroid hormone receptors can be designed as antagonists or selective agonists that completely or partially block receptor functions. For example, anti-androgens are used for prostate cancer therapy, and anti-estrogens are used for breast cancer treatment. GC represents a significant part of blood cancer therapy as well as supportive therapy in solid cancers [35,38,39,40,41,42,43,44,45,46].

Selective hormone receptor modulators may be safer alternatives to classic steroids. In particular, selective glucocorticoid receptor (GR) agonists/modulators (SEGRAMs) of natural and synthetic origin are considered anti-cancer and anti-inflammatory drugs with an improved therapeutic index [22,47,48,49,50,51,52,53,54,55,56,57,58,59,60]. Several SEGRAMs have entered clinical trials, but to date, no drug from the SEGRAM class has reached the pharmaceutical market [61,62,63,64,65,66,67,68]. In our previous studies, we considered Compound A (CpdA) or synephrine, originating from terrestrial plants, as templates for novel SEGRAM synthesis. The mechanism of SEGRAM biological activity is realized via GR binding as standard GCs but lacking GR dimerization, nuclear translocation, and GR-dependent gene transcription (transactivation, TA) associated with GC-related adverse effects. Protein–protein interactions of GR monomers with transcription factors (TFs) mediating therapeutic effects are fully implemented in the SEGRAM activity profile [51,52].

Marine life demonstrates infinite biodiversity, with biologically active low-molecular-weight molecules from the classes of polyketides, alkaloids, terpenoids, polyphenols, and steroids with antimicrobial, anti-cancer, anti-inflammatory, and wound-healing activities [69,70,71,72,73,74,75,76,77,78]. In this review, we aimed to follow marine sources of biologically active steroids and analyze the possibility of finding potential SEGRAMs for cancer treatment. The review is based on the literature available in the PubMed, Cochrane, and web resource ClinicalTrials.gov databases. The review covers the period from 1986 to 2025. The keywords used were “steroids”, “cancer”, and “marine-derived steroids”. The second iteration of the literature search included the keywords “selective glucocorticoid receptor agonists”, “marine-derived”, and “cancer”. In silico calculations of several marine-derived compounds were performed to support the hypothesis based on the literature data.

## 2. Marine-Derived Steroids with Anti-Cancer Activity

To date, the development of novel molecules has demonstrated a reverse shift to molecules of natural origin, particularly from the marine environment. Owing to geographical and topographical peculiarities, the components of marine organisms are not well studied compared to terrestrial organisms; however, technological progress has made it possible to collect organisms from deep-sea water and study their biologically active components [79]. Natural marine products frequently show favorable pharmacokinetic profiles, multiple molecular targets, and a wide spectrum of high biological activities, including anti-inflammatory, antimicrobial, antiviral, wound healing, and anti-cancer effects [27,28,80,81,82,83]. Moreover, marine-derived compounds are characterized by great structural diversity and may include polyketides, terpenoids, alkaloids, steroids, peptides, and others [84,85].

Marine ecosystems, including microorganisms, algae, sea grass, echinoderms, chordates, cnidarians, sponges, and other invertebrates and vertebrates, produce many steroids with significant anti-cancer potential (Table 1). Thus, novel steroids, 5a-cholesta-24-en-3b,20b-diol-23-one (**1**) and 5α-cholesta-9(11)-en-3β,20β-diol (**2**), were isolated from *Acanthaster planci* (crown of thorns starfish) and characterized by anti-cancer activity on MCF-7 breast cancer cells of luminal A subtypes [86]. Steroid dendrodoristerol (**3**) is found in Vietnamese nudibranch *Dendrodoris fumata* and demonstrates cytotoxic effects on a panel of cancer cells of different origins (hepatocellular carcinoma cells HepG2, prostate cancer cells LNCaP, breast cancer cells MCF-7, lung adenocarcinoma cells SK-LU-1, epidermal carcinoma cells KB, leukemia cells HL-60) [87]. Among the compounds isolated from the cold-water starfish *Ctenodiscus crispatus*, the cytotoxic effects of (25S)-5α-cholestane-3β,5,6β,15α,16β,26-hexaol (**4**) against hepatocellular carcinoma cells HepG2 and glioblastoma cells U87MG were reported [88].

(3E)-cholest-4-en-3,6-dione-3-oxime (**5**) from the marine sponge *Cinachyrella australiensis* also exhibited cytotoxic activity against hepatocellular carcinoma cells HepG2 [89]. Other steroid compounds from marine sponges, gracilosulfates A-G (**6**–**10**) from *Haliclona gracilis* species [90], sterols (**11**–**12**) from *Echinoclathria gibbosa* [91], and trihydroxysterols (**13**–**16**) from *Psammoclema* sp. [92], have been demonstrated to inhibit the proliferation of prostate cancer cells 22Rv1, PC-3, and DU-145, respectively.

Two asterosaponins, archasterosides A (**17**) and B (**18**), containing 3β,6α-dihydroxysteroid aglycons with a 9(11)-double bond and sulfate group at C-3, from the starfish *Archaster typicus*, showed moderate anti-cancer activity against cervical cancer cells HeLa [93]. Other asterosaponins and glycosylated steroids were found in starfish from *Culcita novaeguineae*, *Linckia laevigata*, and *Halityle regularis* (**19**–**22**) and exhibited significant cytotoxic effects against prostate cancer cells LNCaP [94]. Spiculiferosides A (**23**), B (**24**), and C (**25**), isolated from the starfish *Henricia leviuscula spiculifera* collected from the Sea of Okhotsk, exhibited weak cytotoxic effects on melanoma SK-MEL-28, breast cancer MDA-MB-231, and colorectal cancer HCT 116 cells. However, they demonstrated the possibility of inducing cell cycle arrest and suppressing colony formation via the inhibition of CDK2, CDK4, cyclins, and MAPK/ERK signaling [95]. Steroidal 3β,21- and 3β,22-disulfates (**26**–**31**), isolated from the Eastern starfish *Pteraster marsippus*, have also been shown to inhibit colony formation of breast cancer cells [96]. Esters of polyhydroxy steroids and long-chain fatty acids (**32**–**35**) from the starfish *Ceramaster patagonicus* have been shown to inhibit the proliferation of breast and colorectal cancer cells and suppress their migratory activity, suggesting the role of these steroids in the therapy of metastatic cancers [97]. (23R)-Methoxycholest-5,24-dien-3β-ol (**36**), isolated from the marine bryozoan *Cryptosula pallasiana*, exhibited cytotoxic effects against leukemia, liver, and gastric cancer [98].

Three ergostane-type steroid compounds from marine-derived fungus *Penicillium levitum*, namely cerevisterol (**37**), ergosterol peroxide (**38**), and (3b,5a,22E)-ergosta-6,8(14),22-triene-3,5-diol (**39**), are characterized by an antiproliferative effect in vitro, and (**39**) is the most potent cytotoxin against cancer cell lines Hep-G2, A549, and MCF-7, while IC_50_ values for (**37**) and (**38**) were not reached [99]. Steroids from marine algae *Tydemania expeditionis*, (E)-stigmasta-24(28)-en-3,6-dione (**40**), fucosterol (**41**), and saringosterol (**42**) demonstrate cytotoxic activity against prostate cancer cells LNCaP, DU-145, and PC-3, with IC_50_ values in the micromolar range [80,100]. Cytotoxic and pro-apoptotic effects realized via ERK1/2-MAPK signaling inactivation in prostate carcinoma PC-3 have also been demonstrated for steroidal constituents (**43**–**54**) from the sea urchin *Diadema savignyi Michelin* [101].

Soft corals represent a distinct class of the marine biosphere and are another source of steroid compounds with potential in cancer treatment. In particular, klyflaccisteroids (**55**–**58**) from the soft coral *Klyxum flaccidum* exhibit cytotoxicity against colon cancer HT-29 cells, lung cancer A549 cells, and murine leukemia P388 [102]. One of the 12 novel steroids found in the soft coral *Sinularia conferta*, ergosta-24(28)-ene-3β,5α,6β-triol-6-acetate (**59**), exhibited a higher cytotoxic effect in lung and cervical cancer cells compared to camptothecin and etoposide [29]. The component of the soft coral *Dendronephthya* species extract, dendronestadione (**60**), significantly inhibits the proliferation of hepatocellular, prostate, and colorectal carcinoma cells in vitro [103]. (22E)-4α,24-dimethyl-5α-cholesta-22,24(28)-dien-3β,8β-diol (**61**) and (22E,24R)-7β-acetoxy-24-methyl-cholesta-5,22-dien-3β,19-diol (**62**) exhibited strong cytotoxic effects on breast cancer cells MCF-7 [104].

In summary, it should be noted that the number of marine-derived steroids with weak or moderate anti-cancer activity in vitro has not been mentioned above. Moreover, many steroids of marine origin have been chemically characterized, but their biological properties, particularly anti-cancer activity, have not yet been tested. And vice versa, many of the tested steroids were studied in the form of total extracts with cytotoxic effects in vitro, but they were not isolated and characterized as individual chemicals [105,106,107,108,109]. To date, none of the steroidal compounds characterized in vitro have entered in vivo preclinical studies or clinical trials, providing a broad field of investigation. Interestingly, hormone-dependent cancers, such as breast and prostate neoplasms, presented as the cancer models most frequently used for cytotoxicity evaluation and confirmation. This provides a rationale for further studies on androgen and estrogen receptor signaling as potential molecular mechanisms of action of marine-derived steroids [110]. In in vitro experiments, blood cancer cells were sensitive to several steroidal compounds, assuming specific cytotoxic activity against lymphocytes and similarity to glucocorticoid effects in hematological malignancies. In the next section, we discuss the possibility of replacing GC-based therapies with marine-derived steroids/ligands of GC receptors.

**Table 1:** Steroids from marine ecosystems with anti-cancer activity.

No.	Name	Structure	Source	Anti-Cancer Activity	Ref.
**1**	5α-cholesta-24-en-3β,20β-diol-23-one	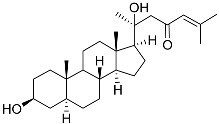	Crown-of-thorns starfish *Acanthaster planci*	Cytotoxic activity against luminal A breast cancer cells MCF-7 in MTT assay IC_50_ 49 ± 1.6 μg/mL	[86]
**2**	5α-cholesta-9(11)-en-3β,20β-diol	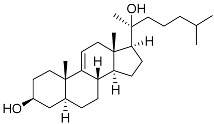	Crown-of-thorns starfish *Acanthaster planci*	Cytotoxic activity against luminal A breast cancer cells MCF-7 in MTT assay IC_50_ 57.5 ± 1.5 μg/mL	[86]
**3**	Dendrodoristerol	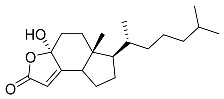	Sea slug *Dendrodoris* *fumata*	Cytotoxic activity against hepatocellular carcinoma cells HepG2, prostate cancer cells LNCaP, breast cancer cells MCF-7, lung adenocarcinoma cells SK-LU-1, epidermal carcinoma cells KB, leukemia cells HL-60 in SRB assay IC_50_ 21.63 ± 2.22, 22.22 ± 1.81, 24.53 ± 2.47, 41.19 ± 3.25, 25.34 ± 3.81, and 21.59 ± 1.38 μM	[87]
**4**	(25S)-5α-cholestane-3β,5,6β,15α,16β,26-hexaol	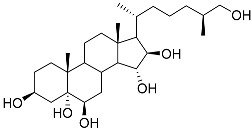	Mud star *Ctenodiscus crispatus*	Shows cytotoxic activity against hepatocellular carcinoma cells HepG2 in MTT assay	[88]
**5**	(3*E*)-cholest-4-en-3,6-dione-3-oxime	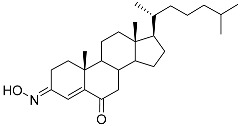	Sea sponge *Cinachyrella australiensis*	Cytotoxic activity against hepatocellular carcinoma cells HepG2 in MTT assay IC_50_ 2.91 mg/mL	[89]
**6**	Gracilosulfate A	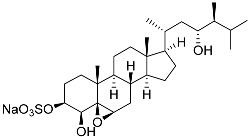	Sea sponge *Haliclona gracilis*	Cytotoxic activity against prostate cancer cell line 22Rv1 in MTT assay IC_50_ 64.4 ± 14.9 μM	[90]
**7**	Gracilosulfate B	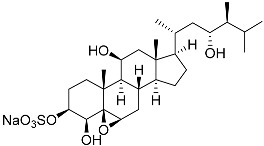	Sea sponge *Haliclona gracilis*	Cytotoxic activity against prostate cancer cell line 22Rv1 in MTT assay IC_50_ > 100 μM	[90]
**8**	Gracilosulfate D	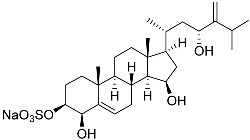	Sea sponge *Haliclona gracilis*	Cytotoxic activity against prostate cancer cell line 22Rv1 in MTT assay IC_50_ > 100 μM	[90]
**9**	Gracilosulfate F	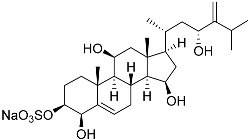	Sea sponge *Haliclona gracilis*	Cytotoxic activity against prostate cancer cell line 22Rv1 in MTT assay IC_50_ > 100 μM	[90]
**10**	Gracilosulfate G	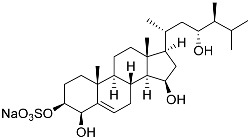	Sea sponge *Haliclona gracilis*	Cytotoxic activity against prostate cancer cell line 22Rv1 in MTT assay IC_50_ > 100 μM	[90]
**11**	β-sitosterol-3-O-(3Z)-pentacosenoate	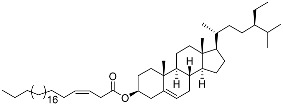	Sea sponge *Echinoclathria gibbosa*	Cytotoxic activity against prostate cancer cells PC-3 in MTT assay IC_50_ 64 μM	[91]
**12**	5α-pregna-3β-acetoxy-12β,16β-diol-20-one	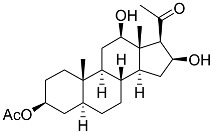	Sea sponge *Echinoclathria gibbosa*	Cytotoxic activity against prostate cancer cells PC-3 in MTT assay IC_50_ > 100 μM	[91]
**13**	3α,12α,16α-trihydroxy-24ξ-ethylcholest-25-ene	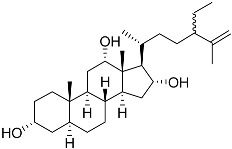	Sea sponge *Psammoclema*	Cytotoxic activity against prostate cancer cells DU-145 in MTT assay GI_50_ 13 ± 1 μM	[92]
**14**	3α,12α,16α-trihydroxy-24R-methylcholest-22E-ene	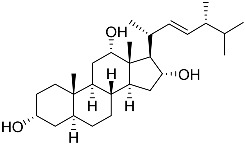	Sea sponge *Psammoclema*	Cytotoxic activity against prostate cancer cells DU-145 in MTT assay GI_50_ 27 ± 1 μM	[92]
**15**	3α,12α,16α-trihydroxy-24-methylcholest-24(28)-ene	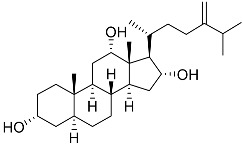	Sea sponge *Psammoclema*	Cytotoxic activity against prostate cancer cells DU-145 in MTT assay GI_50_ 27 ± 1 μM	[92]
**16**	3α,12α,16α-trihydroxycholestane	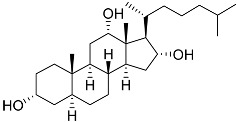	Sea sponge *Psammoclema*	Cytotoxic activity against prostate cancer cells DU-145 in MTT assay GI_50_ 6.7 ± 0.2 μM	[92]
**17**	Archasteroside A	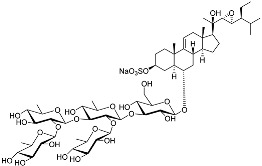			[93]
**18**	Archasteroside B	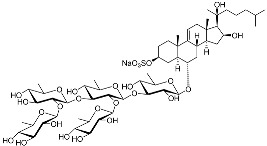			[93]
**19**	Halityloside A	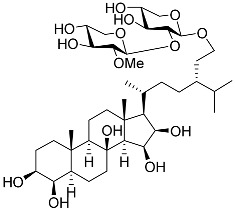	Starfish *Culcita novaeguineae*	Cytotoxic activity against prostate cancer cells LNCaP in SRB assay IC_50_ 48.59 ± 2.30 μM	[94]
**20**	Halityloside B	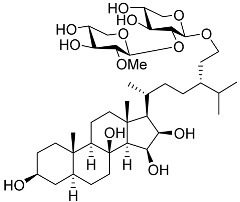	Starfish *Culcita novaeguineae*	Cytotoxic activity against prostate cancer cells LNCaP in SRB assay IC_50_ 39.68 ± 2.65 μM	[94]
**21**	Culcitoside C_5_	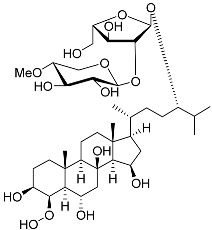	Starfish *Culcita novaeguineae*	Cytotoxic activity against prostate cancer cells LNCaP in SRB assay IC_50_ 57.08 ± 1.81 μM	[94]
**22**	Halityloside D	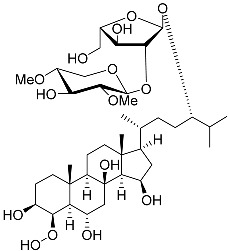	Starfish *Culcita novaeguineae*	Cytotoxic activity against prostate cancer cells LNCaP in SRB assay IC_50_ 31.80 ± 1.59 μM	[94]
**23**	Spiculiferosides A	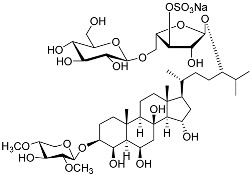	Starfish *Henricia leviuscula spiculifera*	Inhibition of colony formation colorectal carcinoma cells HCT 116 at concentration 40 μM was 65%	[95]
**24**	Spiculiferosides B	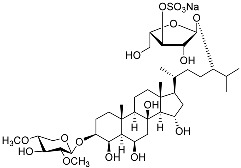	Starfish *Henricia leviuscula spiculifera*	Inhibition of colony formation colorectal carcinoma cells HCT 116 at concentration 40 μM was 81%	[95]
**25**	Spiculiferosides C	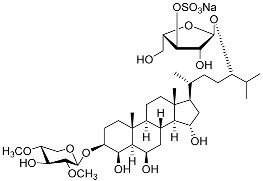	Starfish *Henricia leviuscula spiculifera*	Cytotoxic activity against colorectal carcinoma cells HCT 116 in MTS assay IC_50_ 87.6 μM Inhibition of colony formation colorectal carcinoma cells HCT 116 at concentration 40 μM was 87%	[95]
**26**	(20*R*,22*E*)-24-norcholesta-5,22-diene-3β,21-diol 3,21-disulfate disodium salt	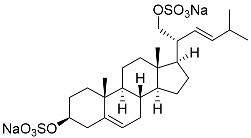	Starfish *Pteraster marsippus*	Inhibition of colony formation breast cancer cells T-47D at concentration 50 μM was 76%	[111]
**27**	(20*R*,22*E*)-24-nor-5α-cholest-22-ene-3β,21-diol 3,21-disulfate disodium salt	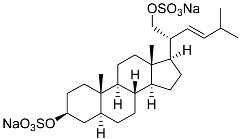	Starfish *Pteraster marsippus*	Inhibition of colony formation breast cancer cells T-47D at concentration 50 μM was 86%	[111]
**28**	(20*R*)-7-oxo-24-methylcholesta-5,24(28)-diene-3β ,21-diyl disulfate disodium salt	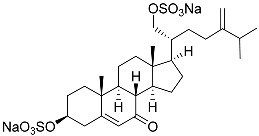	Starfish *Pteraster marsippus*	Cytotoxic activity of the mixture of 28 and 29 against human breast carcinoma cells ZR-75-1 in MTS assay IC_50_ 90.4 μM	[96]
**29**	(20*R*)-7-oxo-24-methyl-5α-cholest-24(28)-ene-3 β,21-diyl disulfate disodium salt	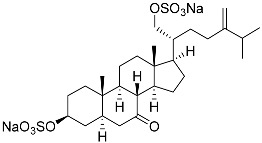	Starfish *Pteraster marsippus*	Cytotoxic activity of the mixture of 28 and 29 against human breast carcinoma cells ZR-75-1 in MTS assay IC_50_ 90.4 μM	[96]
**30**	(20*S*,22*R*)-24-metylcholesta-5,24-diene-3β,22-diol 3,22-disulfate disodium salt	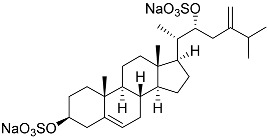	Starfish *Pteraster marsippus*	Inhibition of colony formation breast cancer cells T-47D at concentration 50 μM was 71%	[111]
**31**	(20*S*,22*R*)-24-metyl-5α-cholest-24-ene-2β,3α,22-triol 3,22-disulfate disodium salt	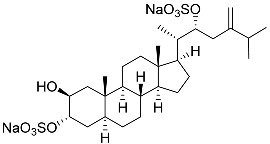	Starfish *Pteraster marsippus*	Inhibition of colony formation breast cancer cells T-47D at concentration 50 μM was 79%	[111]
**32**	(25*S*)-5α-cholestane-3β,6β,15α,16β-tetraol-26-yl 5′*Z*,11′*Z*-octadecadienoate	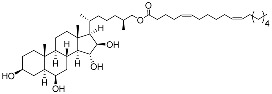	Starfish *Ceramaster patagonicus*	Inhibitory activity against migration of colorectal carcinoma cells HCT 116 was 36%	[97]
**33**	(25*S*)-5α-cholestane-3β,6β,15α,16β-tetraol-26-yl 11’Z-octadecenoate	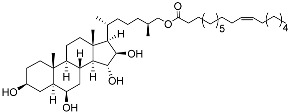	Starfish *Ceramaster patagonicus*	Inhibitory activity against migration of colorectal carcinoma cells HCT 116 was 73%	[97]
**34**	(25*S*)-5α-cholestane-3β,6β,15α,16β-tetraol-26-yl 5’Z,11’Z-eicosadienoate	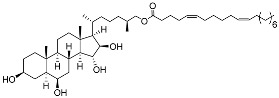	Starfish *Ceramaster patagonicus*	Inhibitory activity against migration of colorectal carcinoma cells HCT 116 was 30%	[97]
**35**	(25*S*)-5α-cholestane-3β,6β,15α,16β-tetraol-26-yl 7’Z-eicosenoate	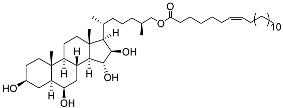	Starfish *Ceramaster patagonicus*	Inhibitory activity against migration of colorectal carcinoma cells HCT 116 was 24%	[97]
**36**	(23*R*)-methoxycholest-5,24-dien-3β-ol	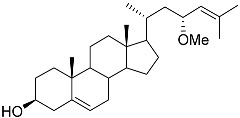	Colonial bryozoan *Cryptosula pallasiana*	Cytotoxic activity against hepatocellular carcinoma cells HepG2, gastric carcinoma cells SGC-7901, and leukemia cells HL-60 in MTT assay IC_50_ 12.34 ± 0.12, 18.37 ± 0.17, and 17.64 ± 0.32 μM	[98]
**37**	Cerevisterol	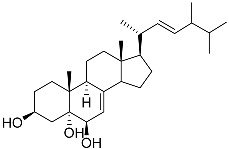	Marine fungus *Penicillium levitum*	Cytotoxic activity against hepatocellular carcinoma cells HepG2, lung carcinoma cells A549, and breast cancer cells MCF-7 in MTT assay was not detected	[99]
**38**	Ergosterol peroxide	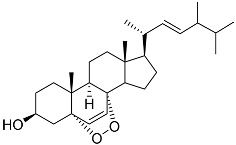	Marine fungus *Penicillium levitum*	Cytotoxic activity against hepatocellular carcinoma cells HepG2, lung carcinoma cells A549, and breast cancer cells MCF-7 in MTT assay IC_50_ 16.22, 22.48, and 27.11 μM	[99]
**39**	(3β,5α,22*E*)-ergosta-6,8(14),22-triene-3,5-diol	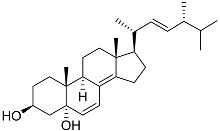	Marine fungus *Penicillium levitum*	Cytotoxic activity against hepatocellular carcinoma cells HepG2, lung carcinoma cells A549, and breast cancer cells MCF-7 in MTT assay IC_50_ 2.89, 18.51, and 16.47 μM	[99]
**40**	(24*E*)-stigmasta-24(28)-en-3,6-dione	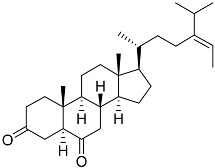	Green algae *Tydemania expeditionis*	Cytotoxic activity against prostate cancer cells DU-145, prostate cancer cells PC-3, and prostate cancer cells LNCaP in MTT assay IC_50_ 31.27 ± 1.50, 40.59 ± 3.10, and 19.80 ± 3.84 μM	[100]
**41**	Fucosterol	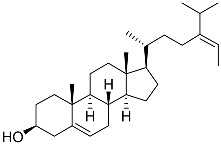	Green algae *Tydemania expeditionis*	Cytotoxic activity against prostate cancer cells DU-145, prostate cancer cells PC-3, and prostate cancer cells LNCaP in MTT assay IC_50_ 12.38 ± 2.47, 2.14 ± 0.33, and 1.38 ± 0.07 μM	[100]
**42**	Saringosterol	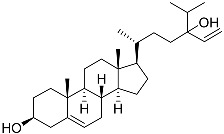	Green algae *Tydemania expeditionis*	Cytotoxic activity against prostate cancer cells DU-145, prostate cancer cells PC-3, and prostate cancer cells LNCaP in MTT assay IC_50_ > 50, > 50, and 41.60 ± 4.26 μM	[100]
**43**	Cholest-8-ene-3β,5α,6β,7α-tetraol	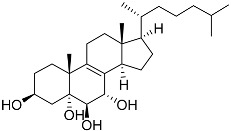	Sea urchin *Diadema savignyi*	Cytotoxic activity against prostate cancer cells PC-3 in MTT assay IC_50_ 40.43 ± 1.45 μM	[101]
**44**	Cholest-8(14)-ene-3β,5α,6β,7α-tetraol	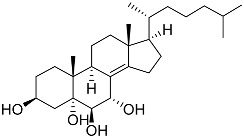	Sea urchin *Diadema savignyi*	Cytotoxic activity against prostate cancer cells PC-3 in MTT assay IC_50_ 5.49 ± 0.22 μM	[101]
**45**	Cholest-7-ene-3β,5α,6β-triol	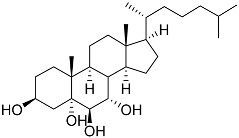	Sea urchin *Diadema savignyi*	Cytotoxic activity against prostate cancer cells PC-3 in MTT assay IC_50_ 74.06 ± 3.46 μM	[101]
**46**	Cholest-7-ene-3β,5α,6α,9α-tetraol	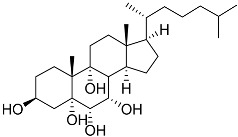	Sea urchin *Diadema savignyi*	Cytotoxic activity against prostate cancer cells PC-3 in MTT assay IC_50_ 27.41 ± 0.50 μM	[101]
**47**	Cholest-7-ene-6-one-3β,5α,9α-triol	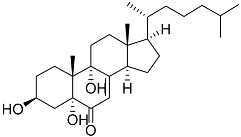	Sea urchin *Diadema savignyi*	Cytotoxic activity against prostate cancer cells PC-3 in MTT assay IC_50_ 24.40 ± 0.46 μM	[101]
**48**	Cholest-5-ene-3β,7α-diol	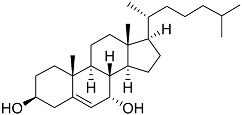	Sea urchin *Diadema savignyi*	Cytotoxic activity against prostate cancer cells PC-3 in MTT assay IC_50_ 29.22 ± 0.17 μM	[101]
**49**	Cholest-5-ene-3β,7β-diol	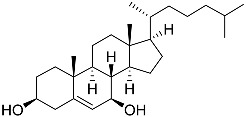	Sea urchin *Diadema savignyi*	Cytotoxic activity against prostate cancer cells PC-3 in MTT assay IC_50_ 27.94 ± 0.63 μM	[101]
**50**	Cholest-5-ene-7β-methoxy-3β-ol	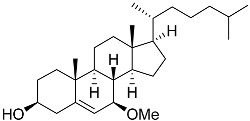	Sea urchin *Diadema savignyi*	Cytotoxic activity against prostate cancer cells PC-3 in MTT assay IC_50_ 9.22 ± 0.67 μM	[101]
**51**	Campesterol	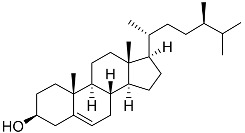	Sea urchin *Diadema savignyi*	Cytotoxic activity against prostate cancer cells PC-3 in MTT assay IC_50_ 22.26 ± 0.59 μM	[101]
**52**	Cholest-5-ene-3β-sulfate sodium solt	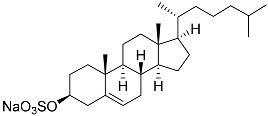	Sea urchin *Diadema savignyi*	Cytotoxic activity against prostate cancer cells PC-3 in MTT assay IC_50_ 68.87 ± 6.08 μM	[101]
**53**	Cholest-6-ene-5α,8α-epidioxy-3β-ol	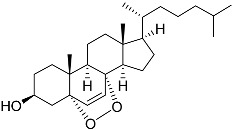	Sea urchin *Diadema savignyi*	Cytotoxic activity against prostate cancer cells PC-3 in MTT assay IC_50_ 6.99 ± 0.28 μM	[101]
**54**	Cholest-5-ene-3β-ol	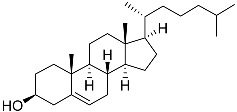	Sea urchin *Diadema savignyi*	Cytotoxic activity against prostate cancer cells PC-3 in MTT assay was not detected	[101]
**55**	Klyflaccisteroid A	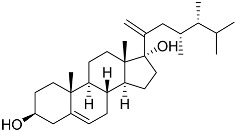	Soft coral *Klyxum flaccidum*	Cytotoxic activity against colon cancer cells HT-29, lung cancer cells A549, and murine leukemia cells P388 in Alamar Blue assay ED_50_ > 20, 7.7, and >20 μg mL^−1^	[102]
**56**	Klyflaccisteroid F	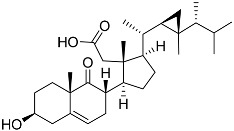	Soft coral *Klyxum flaccidum*	Cytotoxic activity against colon cancer cells HT-29, lung cancer cells A549, and murine leukemia cells P388 in Alamar Blue assay ED50 > 20, 14.5, and 17.9 μg mL^−1^	[102]
**57**	Klyflaccisteroid C	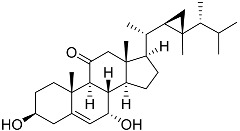	Soft coral *Klyxum flaccidum*	Cytotoxic activity against colon cancer cells HT-29, lung cancer cells A549, and murine leukemia cells P388 in Alamar Blue assay ED_50_ 8.2, 6.1, and 10.8 μg mL^−1^	[102]
**58**	Klyflaccisteroid E	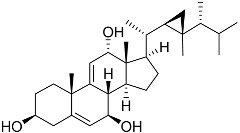	Soft coral *Klyxum flaccidum*	Cytotoxic activity against colon cancer cells HT-29 and murine leukemia cells P388 in Alamar Blue assay ED_50_ 6.9 and 3.7 μg mL^−1^	[102]
**59**	Ergosta-24(28)-ene-3β,5α,6β-triol-6-acetate	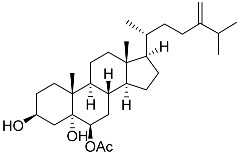	Soft coral *Sinularia conferta*	Cytotoxic activity against lung cancer cells A549, cervical adenocarcinoma cells HeLa, and pancreatic epithelioid carcinoma cells PANC-1 in MTT assay IC_50_ 3.64 ± 0.18, 19.34 ± 0.42, and 1.78 ± 0.69 μM	[112]
**60**	Dendronestadione	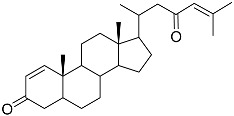	Soft coral *Dendronephthya*	Cytotoxic activity against hepatocellular carcinoma cells HepG2, colon cancer cells HT-29, and prostate cancer cells PC-3 in MTT assay IC_50_ 19.1 ± 1.81, 32.4 ± 2.84, and 7.8 ± 0.80 μM	[113]
**61**	(22*E*)-4α,24-dimethyl-5α-cholesta-22,24(28)-dien-3β,8β-diol	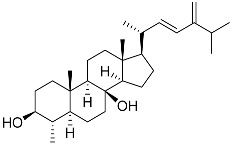	Soft coral *Litophyton mollis*	Cytotoxic activity against hepatocellular carcinoma cells HepG2, breast cancer cells MCF-7, and lung carcinoma cells NCI-H1299 in SRB assay IC_50_ > 50 μM in all cases	[104]
**62**	(22*E*,24*R*)-7β-acetoxy-24-methylcholesta-5,22-dien-3β,19-diol	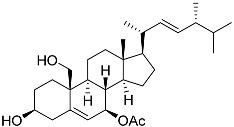	Soft coral *Litophyton mollis*	Cytotoxic activity against hepatocellular carcinoma cells HepG2, breast cancer cells MCF-7, and lung carcinoma cells NCI-H1299 in SRB assay IC_50_ 32.5, 8.4, and 15.1 μM	[104]

## 3. Potential Glucocorticoid Receptor Modulators from Natural Marine Products

To discuss the role of GC in cancer therapy, the signaling of the glucocorticoid receptor (GR), a well-known transcription factor (TF) and mediator of GC biological effects in cells and tissues, should be briefly explained. Binding of GC to inactive GR in the cytoplasm leads to receptor activation, homodimerization, and translocation to the nucleus. The GR-GR homodimer binds to GC-responsive elements (GREs) in DNA, resulting in induction or inhibition of the transcription of different gene subsets. Protein–protein interactions of GR monomers with other TFs are followed by the suppression of their activity [114,115]. GR-dependent inhibition of pro-proliferative and anti-apoptotic TF activity or suppression of gene transcription (transrepression, TR) mediates the therapeutic effects of GC. Induction of GR-dependent gene transcription (transactivation, TA) is mainly associated with metabolic and atrophic GC-related complications [116,117]. Therefore, the development of GC analogs of synthetic or natural origin with an improved therapeutic index and attenuated side effects is of interest, with the compounds of the SEGRAM class as an option [50,118,119,120].

GR is a member of the nuclear receptor superfamily, which also includes the estrogen receptor (ER), progesterone receptor (PR), androgen receptor (AR), mineralocorticoid receptor (MR), vitamin D receptor (VDR), and thyroid hormone receptor (ThR). Steroid receptors have a highly conserved DNA-binding domain (DBD), which allows them to bind to the responsive elements of other family members [121,122]. It could mediate the glucocorticoid-like activity of potential ER, PR, or AR ligands, and vice versa. Thus, GR can form a heterodimer with AR, modulating its activity [123,124,125,126]. The homology of GR and PR DBDs allows the sharing of responsive elements and regulation of the expression of immunophilins, oncogenes, and TFs [127,128]. Cross-talk between GR and ER occurs via protein–protein interactions of receptor monomers, followed by binding of the heterodimer to estrogen-responsive elements (EREs) as well as direct suppression of ER activity. This interaction could explain the anti-proliferative activity of GC in ER-positive breast cancer cells [129,130,131,132]. However, it should be noted that ER-GR interactions may lead to breast cancer progression and metastasis in ER-negative cancer subtypes [133,134,135,136].

To date, little is known about the GR-dependent anti-cancer activity of marine-derived steroids. Gene expression profiles in GC anti-inflammatory and anti-cancer effects significantly intersect, allowing marine-derived steroids with anti-inflammatory properties to be considered as a potential option for cancer treatment (Table 2). Thus, klyflaccisteroids from soft corals (**56**, **57**) with cytotoxic potential also exhibit strong anti-inflammatory effects in vitro, specifically the inhibition of superoxide anion generation and elastase release in human neutrophils [102]. The fungus *Penicillium oxalicum*, associated with the soft coral *Sinularia gaweli*, produces ergostane-type sterol ester (**63**). This sterol ester demonstrated anti-inflammatory activity in RAW264.7 macrophage cells by inhibiting the expression of pro-inflammatory cytokines TNF-α and INF-β1 [137]. (22E,24R)-ergosta-5,7,22-trien-3β-ol (**64**), isolated from the *Avicennia* mangrove-associated marine fungus *Amorosia* sp., suppressed LPS-induced NO production and pro-inflammatory factors IL-6, TNF-α, and MCP-1. However, no cytotoxic activity was observed [138]. Ergosterol (**65**), found in the deep-sea fungus *Samsoniella hepiali*, inhibits NO production in LPS-activated microglia cells [139]. Similar inhibitory effects on the inflammation markers iNOS, TNF-α, IL-6, and IL-1β, at both the mRNA and protein levels in vitro, have been described for sesquiterpenoid (**66**) isolated from the marine-derived fungus *Eutypella* sp. [140], persteroid (**67**) isolated from the marine-derived fungus *Penicillium* sp. ZYX-Z-143 [141], and ergostane-type steroid components (**37**–**39**) from the marine-derived fungus *Penicillium levitum* with cytotoxic potential against breast, lung, and liver cancer cells [99]. Non-steroidal components of a marine-derived actinomycete strain, identified as a *Streptomyces* sp., taking the form of ten new nine-membered *bis*-lactones, splenocins A-J (**68**–**77**), with anti-inflammatory activity compared to GC dexamethasone in a splenocyte cytokine assay, were also described in the literature [142].

The most intriguing case in the reviewed subsets of marine-derived steroids is fucosterol (**41**), which has been reported to exhibit anti-cancer activity in vitro in prostate and breast cancer cells. Molecular docking of fucosterol on LXR-β, GR, TrkB, TLR2/4, BACE1, and AChE showed that fucosterol formed several hydrophobic interactions with GR via Met604, Leu608, and Phe623. The reported molecular docking data on fucosterol’s GR-binding affinity also suggest its anti-inflammatory action [143]. Following the observation that fucosterol decreases angiotensin-converting enzyme (ACE) levels in endothelial cells by inhibiting GR synthesis involved in ACE regulation [144], the interaction mode of fucosterol with GR could be considered antagonism, but further studies are needed.

Thus, the possibility of interaction with GR has been described only for one marine steroid, despite the fact that more than 1000 marine steroids, including those isolated from marine fungi and sponges, are currently known. We calculated in silico the GR interaction with some marine sponge- and fungal-derived steroidal compounds, which were previously investigated by one of us, to propose their GR binding (Table 3). Molecular docking evaluation is described in detail in [145]. The structure of GR (PDB ID 1P93) was obtained from the RCSB Protein Data Bank (https://www.rcsb.org, accessed on 27 August 2025).

3β,15β-Dihydroxy-(22*E*,24*R*)-ergosta-5,8(14),22-trien-7-one (**78**) has been isolated from Vietnamese marine fungus *Penicillium chermesinum* 2104NT-1.3 and reported as a cardioprotective agent [110]. Molecular docking calculations showed that (**78**) did not interact with the ligand-binding domain (LBD) of the GR. A number of new oxygenated sterol derivatives have been isolated from the marine sponge *Inflatella* sp., collected from the Sea of Okhotsk [146]. 24-Methylcholesta-5,24(28)-diene-3β,4α-diol (**79**) was not active against 6-hydroxydopamine (6-OHDA) toxicity, and 24-*nor*-cholesta-5,22-diene-3β,7α-diol (**80**) increased the 6-OHDA-treated Neuro-2a cell viability. Moreover, (**80**) enhanced formazan production in the MTT assay. The results indicated that (**79**) can interact with Arg611, and (**80**) interacts with Met604, Leu566, Tyr735, Phe623, and Cys736, which form LBD-GR.

Moreover, we attempted to determine whether marine non-steroidal compounds are of interest in this regard. The library of secondary metabolites isolated at various times from the fungal strains of the Collection of Marine Microorganisms of the PIBOC FEB RAS (https://kmm644.ru, accessed on 27 August 2025) was analyzed from the point of view of structural similarity with dexamethasone using the PubChem Score Matrix Service to calculated substructure key-based 2D Tanimoto similarity (https://pubchem.ncbi.nlm.nih.gov/docs/score-matrix-service (accessed on 30 August 2025)). In total, 202 compounds were analyzed, and the highest Tanimoto index of 69 was calculated for decumbenone C (**81**) from *Aspergillus sulphureus* KMM 4640 [147] and conidiogenone F (**82**) from *Penicillium antarcticum* KMM 4670 [148]. The list of compounds and full PubChem Score Matrix calculations are presented in Supplementary File S1.

**Figure 1:**
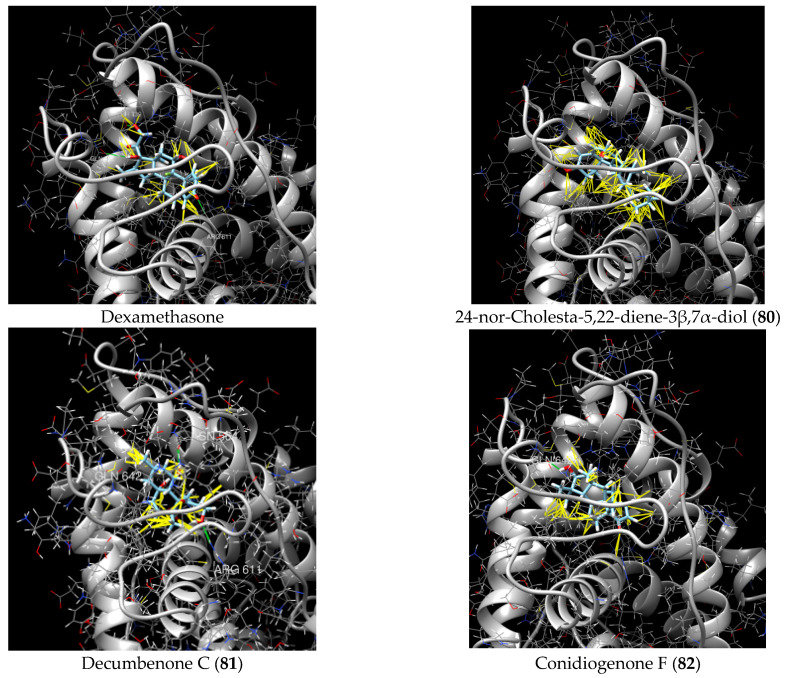
Complexes of LBD-GR with compounds (PDB ID 1P93). Green lines indicate hydrogenic bonds, and yellow lines indicate hydrophobic interactions.

Molecular docking calculations were also performed for these compounds (Figure 1, Table 3), and they showed a good possibility of binding to LBD-GR. Decumbenone C (**81**) formed a complex with ΔG of -8.23 kcal/mol and interacted with Arg611, Gly567, Trp600, Met604, Met601, Met646, Phe623, and Met560. Two complexes were calculated for conidiogenone F (**82**). The first complex, with a ΔG of −8.46 kcal/mol, included hydrophobic interactions with Gly567, Met604, Met601, Trp600, Cys736, Tyr735, Met560, Met646, and Phe623 in LBD-GR. The second complex, with a ΔG of −7.91 kcal/mol, involved hydrogen binding with Gln642 and hydrophobic interactions with Met560, Gly567, Met604, Met646, and Cys736 in LBD-GR. Figure 1 illustrates the complexes of LBD-GR with compounds exhibiting the highest affinity.

**Table 2:** Anti-inflammatory marine-derived compounds of steroidal and non-steroidal structures.

No.	Name	Structure	Source	Anti-Inflammatory Effects	Reference
**56**	Klyflaccisteroid F	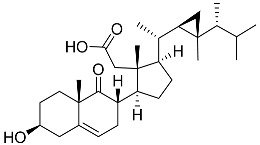	Soft coral *Klyxum flaccidum*	Activity in inhibiting the superoxide anion generation 88.26 ± 35.86% at 10 μM and activity in inhibiting elastase release 104.22 ± 6.55% at 10 μM in N-formyl-methionyl-leucyl-phenylalanine/ cytochalasin B (fMLP/CB)-induced neutrophils	[102]
**57**	Klyflaccisteroid C	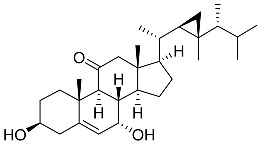	Soft coral *Klyxum flaccidum*	Activity in inhibiting the superoxide anion generation 76.24 ± 5.64% at 10 μM and activity in inhibiting elastase release 88.38 ± 1.19% at 10 μM in N-formyl-methionyl-leucyl-phenylalanine/ cytochalasin B (fMLP/CB)-induced neutrophils	[102]
**63**	(22E,24S)-9a,15a-dihydroxyergosta-4,6,8(14),22- tetraen-3-one 15-palmitate	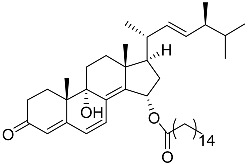	Marine fungus *Penicillium oxalicum* HL-44	Inhibition of expression of pro-inflammatory cytokines TNF-α and INF-β1 on DMXAA-stimulated Raw264.7 cells by 68% and 94% at 20 μM	[137]
**64**	(22E, 24R)-ergosta-5,7,22-trien-3β-ol	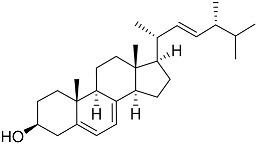	Mangrove fungus *Amorosia* sp.	Inhibition of expression of pro-inflammatory cytokines TNF-α, IL-6, and MCP-1 on LPS-activated RAW264.7 M1-type cells by 55%, 50%, and 50% at 10 μM	[138]
**65**	Ergosterol	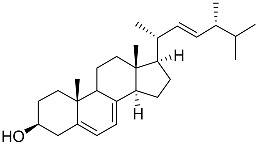	Marine fungus *Samsoniella hepiali* W7	Inhibition of NO production in LPS-activated BV-2-microglia cells by 32.9 ± 1.6% at 1 μM	[139]
**66**	Arctiol	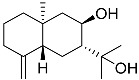	Marine fungus *Eutypella* sp. F0219	Inhibition of NO production in LPS-treated BV-2-microglia cells by 71% at 20 μM	[140]
**67**	Persteroid	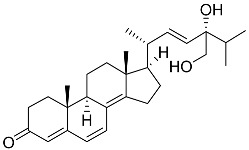	Marine fungus *Penicillium* sp. ZYX-Z-143	NO half-maximal inhibitory concentration on LPS-stimulated RAW 264.7 cells IC_50_ 25.81 ± 0.92 μM	[141]
**37**	Cerevisterol	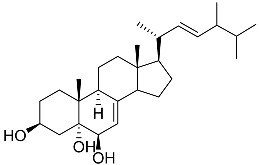	Marine fungus *Penicillium levitum*	NO half-maximal inhibitory concentration on LPS-stimulated RAW 264.7 cells IC_50_ 25.45 μg/mL	[99]
**38**	Ergosterol peroxide	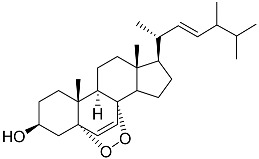	Marine fungus *Penicillium levitum*	NO half-maximal inhibitory concentration on LPS-stimulated RAW 264.7 cells IC_50_ 2.85 μg/mL	[99]
**39**	(3β,5α,22*E*)-ergosta-6,8(14),22-triene-3,5-diol	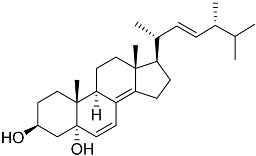	Marine fungus *Penicillium levitum*	NO half-maximal inhibitory concentration on LPS-stimulated RAW 264.7 cells IC_50_ 2.79 μg/mL	[99]
**68**	Splenocin A	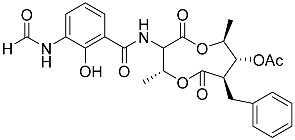	Marine bacterium *Streptomyces* sp.	Inhibition of expression of pro-inflammatory cytokines IL-5 on T_H_^2^ cells (helper T lymphocytes) IC_50_ 3.1 ± 1.2 nM	[142]
**69**	Splenocin B	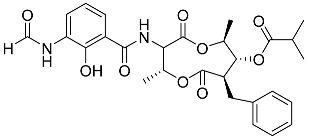	Marine bacterium *Streptomyces* sp.	Inhibition of expression of pro-inflammatory cytokines IL-5 and IL-13 on T_H_^2^ cells (helper T lymphocytes) IC_50_ 1.8 ± 0.2 and 1.6 ± 0.02 nM	[142]
**70**	Splenocin C	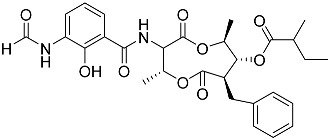	Marine bacterium *Streptomyces* sp.	Inhibition of expression of pro-inflammatory cytokines IL-5 and IL-13 on T_H_^2^ cells (helper T lymphocytes) IC_50_ 6.7 ± 0.2 and 7.3 ± 4.2 nM	[142]
**71**	Splenocin D	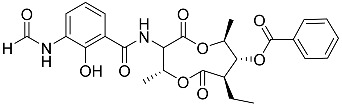	Marine bacterium *Streptomyces* sp.	Inhibition of expression of pro-inflammatory cytokines IL-5 and IL-13 on T_H_^2^ cells (helper T lymphocytes) IC_50_ 47.9 ± 2.9 and 43.7 ± 3.5 nM	[142]
**72**	Splenocin E	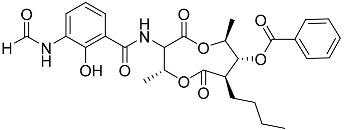	Marine bacterium *Streptomyces* sp.	Inhibition of expression of pro-inflammatory cytokines IL-5 and IL-13 on T_H_^2^ cells (helper T lymphocytes) IC_50_ 16.6 ± 1.8 and 15.9 ± 1.1 nM	[142]
**73**	Splenocin F	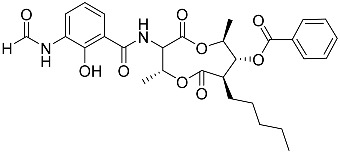	Marine bacterium *Streptomyces* sp.	Inhibition of expression of pro-inflammatory cytokines IL-5 and IL-13 on T_H_^2^ cells (helper T lymphocytes) IC_50_ 9.4 ± 2.8 and 6.8 ± 0.3 nM	[142]
**74**	Splenocin G	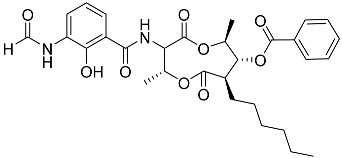	Marine bacterium *Streptomyces* sp.	Inhibition of expression of pro-inflammatory cytokines IL-5 and IL-13 on T_H_^2^ cells (helper T lymphocytes) IC_50_ 5 ± 0.4 and 5.2 ± 0.1 nM	[142]
**75**	Splenocin H	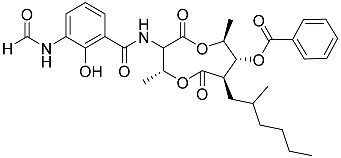	Marine bacterium *Streptomyces* sp.	Inhibition of expression of pro-inflammatory cytokines IL-5 and IL-13 on T_H_^2^ cells (helper T lymphocytes) IC_50_ 4.3 ± 0.5 and 5.1 ± 0.1 nM	[142]
**76**	Splenocin I	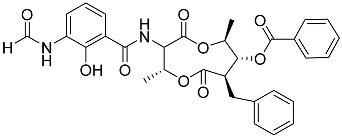	Marine bacterium *Streptomyces* sp.	Inhibition of expression of pro-inflammatory cytokines IL-5 and IL-13 on T_H_^2^ cells (helper T lymphocytes) IC_50_ 15.8 ± 1.0 and 15.2 ± 1.3 nM	[142]
**77**	Splenocin J	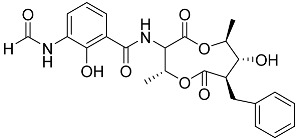	Marine bacterium *Streptomyces* sp.	Inhibition of expression of pro-inflammatory cytokines IL-5 and IL-13 on T_H_^2^ cells (helper T lymphocytes) IC_50_ 1022.7 ± 52.3 and 826.3 ± 187.6 nM	[142]
**41**	Fucosterol	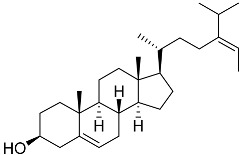	Green algae *Tydemania expeditionis*	Hydrophobic interaction with GR via Leu563, Phe623, Leu608, and Met604 (molecular docking analysis)	[143]

Interestingly, decaline polyketide decumbenone C (**81**) exhibited potent cytotoxic activity against SK-MEL-5 human melanoma cells, with an IC_50_ of 0.9 µM, and inhibited colony formation at 0.25 µM [147]. Cyclopiane diterpene conidiogenone F (**82**) exhibited weak antimicrobial activity and was non-toxic to H9c2 cardiomyocytes [148]. However, its bioactivity has not been studied in detail because of difficulties in isolating it from fungal extracts.

**Table 3:** Molecular docking calculations of natural marine compounds with GR (PDB ID 1P93).

No.	Compound	Chemical Structure	ΔG, kcal/mol	FF Score, kcal/mol	H-Binding	Hydrophobic Interactions
**78**	3β,15β-Dihydroxy-(22*E*, 24*R*)-ergosta-5,8(14),22-trien-7-one	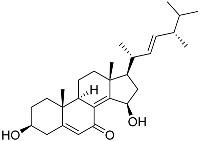	−7.23	−1226.71	-	Val538, ILe539, Lys576, Ala573, Leu544, Trp577
**79**	24-Methylcholesta-5,24(28)-diene-3β,4α-diol	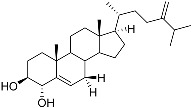	−7.40	−1204.67	H29 Arg611 2.607	Val543, Trp610, Tyr660
**80**	24-*nor*-Cholesta-5,22-diene-3β,7α-diol	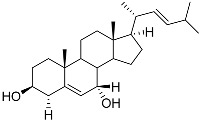	−6.37	−1165.36	-	Met604, Leu566, Leu732, Asn630, Leu563, Tyr735, Phe623, Leu608, Cys736
**81**	Decumbenone C	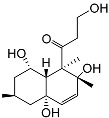	−8.23	−1298.75	H29 Arg611 O 2.666 H24 Asn564 1.682	Gly567, Trp600, Met604, Met601, Leu732, Met646, Phe623, Leu563, Met560
**82**	Conidiogenone F	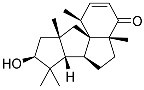	−8.46	−1229.36	-	Gly567, Met604, Met601, Leu732, Trp600, Cys736, Tyr735, Met560, Leu563, Met646, Phe623
			−7.91	−1211.54	H29 Gln642 2.103	Met560, Leu563, Leu753, Gly567, Met604, Met646, Leu732, Cys736
	Dexamethasone	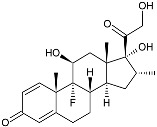	−10.19	−1206.31	H29 Arg611 O 2.146 H27 Gln642 2.351 H26 Thr739 2.115	Met560, Leu566, Gly567, Trp600, Met601, Met604, Phe623, Met646, Tyr735, Cys736, Thr739, Ile747

Structural similarity analysis involves the study of the presence of substructural key elements, along with the distance between pharmacophores or functional groups in compounds; at the same time, the detailed structure and biogenetic origin are not taken into account. This allowed us to select decalin polyketide (**81**) and diterpene derivative (**82**) as compounds structurally similar to dexamethasone, and modular docking confirmed their prospects as GR ligands. Moreover, 10 marine fungal metabolites had Tanimoto indexes in the range of 52–66, and 30 compounds had Tanimoto indexes in the range of 41–49 (Supplementary File S1); these 40 compounds may also be promising GR ligands. We propose that the structural similarity of non-steroidal compounds to steroids may ensure their biological activity but reduce steroid-dependent side effects. This clearly requires additional in-depth research.

## 4. Conclusions

In conclusion, it should be mentioned that marine ecosystems represent a significant source of natural steroids with potential applications in anti-cancer and anti-inflammatory therapies. More specifically, ligands of glucocorticoid receptors and putative selective glucocorticoid receptor agonists/modulators can be found among marine-derived compounds and can be characterized by their chemical structure and biological activity. Evaluation of the affinity to the glucocorticoid receptor and the assessment of the expression of marker genes specific for glucocorticoid receptor activity could be a useful set of methods for the screening of biological activity. The previously published review paper cited in the present manuscript primarily aimed to summarize marine-derived steroids with various biological effects. The novelty of the present review lies not only in highlighting the dual anti-cancer and anti-inflammatory effects mimicking the action of the specific class of steroids, glucocorticoids, but also in the description of the glucocorticoid receptor affinity of several compounds available in the Collection of Marine Microorganisms of the PIBOC FEB RAS. Future directions in this field could involve the experimental validation of the biological effects of the compounds presented in this review and the study of the molecular mechanisms underlying their action. The lead compounds are assumed to bind to the glucocorticoid receptor, revealing anti-cancer and anti-inflammatory effects and, ideally, a “dissociated” activity profile with a shift towards GR TR. In the case of successful proof-of-concept studies, efforts could be made to develop a finished dosage form of the proposed SEGRAMs, followed by preclinical studies of the safety and efficacy of the leader compounds, with further design of first-in-human clinical trials. The results of this study could have translational potential not only in terms of cancer therapy but also for the treatment of inflammatory and autoimmune diseases, which affect millions of patients worldwide.

## Data Availability

Not applicable.

## Supplementary Materials

The following supporting information can be downloaded at https://www.mdpi.com/article/10.3390/md23100399/s1, Table S1. The list of metabolites isolated from fungal strains of the Collection of Marine Microorganisms PIBOC FEB RAS [149,150,151,152,153,154,155,156,157,158,159,160,161,162,163,164].

## Abbreviations

The following abbreviations are used in this manuscript:

ACE	Angiotensin-Converting Enzyme
AChE	Acetyl Cholinesterase
AR	Androgen Receptor
BACE1	Beta-Secretase 1
CDK	Cyclin-Dependent Kinase
CpdA	Compound A
DBD	DNA-Binding Domain
ER	Estrogen Receptor
ERE	Estrogen Response Element
ERK	Extracellular-Regulated Kinase
GC	Glucocorticoid
GR	Glucocorticoid Receptor
GRE	Glucocorticoid Response Element
IL	Interleukin
INF	Interferon
iNOS	Inducible Nitric Oxide Synthase
LPS	Lipopolysaccharide
LXR-β	Liver X Receptor Beta
MAPK	Mitogen-Activated Protein Kinase
MCP-1	Monocyte Chemoattractant Protein-1
MR	Mineralocorticoid Receptor
PR	Progesterone Receptor
SEGRAMs	Selective Glucocorticoid Receptor Agonists/Modulators
TA	Transactivation
TF	Transcription Factor
ThR	Thyroid Hormone Receptor
TLR	Toll-Like Receptor
TNF-α	Tumor Necrosis Factor Alpha
TR	Transrepression
TrkB	Tropomyosin Receptor Kinase B
VDR	Vitamin D Receptor
