# Supplementary material for: Marine-Derived Steroids for Cancer Treatment: Search for Potential Selective Glucocorticoid Receptor Agonists/Modulators (SEGRAMs)
Source: Mar Drugs. 2025 Oct 14;23(10):399. doi: 10.3390/md23100399 (PMC12565360; doi:10.3390/md23100399)
Supplement: Supplementary file 1 [file marinedrugs-23-00399-s001.zip › marinedrugs-3900009-supplementary.pdf]

Table S1. The list of metabolites isolated from fungal strains of the Collection of Marine Microorganisms PIBOC FEB RAS

PubChemID	Compound	2D Similarity to Dexamethasone	Reference
5743	Dexamethasone	Tanimoto index	
**71528821**	**Decumbenone C**	**69**	[148]
**25223313**	**Conidiogenone F**	**69**	[149]
44480472	Ustusolate E	66	[75]
23955806	Strobilactone A	65	[150]
98789427	Asperflavinoid E	59	[75]
38347531	Piltunine A	56	[151]
128296	Pereniporin A	55	[150]
90681916	Oxirapentyn K	54	[152]
71551230	Coniothyrinone D	53	[153]
71551168	Coniothyrinone B	53	[153]
45359249	Radiclonic acid	53	[154]
10633702	Guaiadiol A	52	[155]
177382	Anserinone B	49	[156]
442299	Quadrone	49	[157]
90681914	Oxirapentyn I	48	[152]
38351305	12-Hydroxysydonic acid	48	[154]
90681913	Oxirapentyn F	47	[152]
90681915	Oxirapentyn J	47	[152]
85503103	Carnemycin B	45	[150]
22296216	Aversin	45	[154]
160717	Questin	45	[157]
38351290	Aspilactonol G	44	[158]
443853	4-Hydroxy-3-(3-methylbut-2-enyl)benzaldehyde	44	[157]
160505	Sulochrin	44	[159]
119037	Flavoglaucin	44	[160]
14355115	Isodihydroauroglaucin	44	[160]
13990016	Cladosporin	44	[151]
176918	Rubrofusarine B	44	[161]
146357	Dianhydro-aurasperone C	44	[161]
5280389	Sterigmatocystin	43	[154]
16196967	Dihydroaspirone	43	[158]
10410482	Insulicolide A	43	[158]
10679779	(−)-3-Butyl-7-hydroxyphthalide	43	[162]
3084216	Aurasperone A	43	[161]
11114482	Asperpyrone B	43	[161]
24011606	Candidusin A	42	[163]
5281657	Norlichexanthone	42	[164]
5377910	Griseoxanthone C	42	[164]
179522	Aurasperone B	42	[161]
60158905	Aurasperone F	42	[161]

75069340	5-Hydroxy-6-(3-methylbut-2-enyl)-2-(pent-1-enyl)benzofuran-4-carbaldehyde	41	[160]
72696571	Diorcinol E	41	[165]
